# Violence against women from partners and other household members during COVID-19 in Burkina Faso and Kenya

**DOI:** 10.1186/s12889-022-14197-3

**Published:** 2022-10-05

**Authors:** Michele R. Decker, Shannon N. Wood, Haley L. Thomas, Mary Thiongo, Georges Guiella, Bazie Fiacre, Yentéma Onadja, Peter Gichangi

**Affiliations:** 1 Department of Population, Family and Reproductive Health, Johns Hopkins Bloomberg School of Public Health, 615 N Wolfe St, E4142, Baltimore, MD 21205 USA; 2 Bill & Melinda Gates Institute for Population and Reproductive Health, Department of Population, Family and Reproductive Health, Johns Hopkins Bloomberg School of Public Health, Baltimore, MD USA; 3 International Centre for Reproductive Health-Kenya, Nairobi, Kenya; 4 Institut Supérieur des Sciences de la Population (ISSP/Université Joseph Ki-Zerbo), Ouagadougou, Burkina Faso; 5 Technical University of Mombasa, Mombasa, Kenya; 6 Department of Public Health and Primary Care, Faculty of Medicine and Health Sciences, Ghent University, Ghent, Belgium

**Keywords:** Gender-based violence, Intimate partner violence, Household abuse, Help-seeking, COVID-19, Kenya, Burkina Faso

## Abstract

**Background:**

Global evidence indicates increases in gender-based violence (GBV) during the COVID-19 pandemic following mitigation measures, such as stay at home orders. Indirect effects of the pandemic, including income loss, strained social support, and closed or inaccessible violence response services, may further exacerbate GBV and undermine help-seeking. In Kenya and Burkina Faso, as in many settings, GBV was prevalent prior to the COVID-19 pandemic. Studies specific to COVID-impact on GBV in Kenya indicate mixed results and there remains a lack of evidence from Burkina Faso. Our study takes a comprehensive lens by addressing both intimate partner violence (IPV) and non-partner household abuse through the COVID-19 pandemic in two priority settings.

**Methods:**

Annual, national cross-sections of women ages 15–49 completed survey data collection in November–December 2020 and December 2020–March 2021; the GBV module was limited to one woman per household [Kenya *n* = 6715; Burkina *n* = 4065]. Descriptive statistics, Venn diagrams, and logistic and multinomial regression characterized prevalence of IPV and other household abuse, frequency relative to the COVID-19 pandemic, help-seeking behaviors, and predictors of IPV and household abuse across the socioecological framework.

**Results:**

In both settings, past-year IPV prevalence exceeded non-partner household abuse (Kenya: 23.5%_IPV,_ 11.0%_household_; Burkina Faso: 25.7%_IPV,_ 16.2%_household_). Over half of those affected in each setting did not seek help; those that did turned first to family. Among those with past-year experiences, increased frequency since COVID-19 was noted for IPV (16.0%_Burkina Faso_; 33.6%_Kenya_) and household violence (14.3%_Burkina Faso_; 26.2%_Kenya_). Both context-specific (i.e., financial autonomy in Burkina Faso) and universal (i.e., COVID-related income loss) risk factors emerged.

**Conclusion:**

Past-year IPV and household violence against women in Kenya and Burkina Faso were prevalent, and in some cases, intensified during the COVID-19 pandemic. Across settings, help-seeking from formal services was notably low, likely reflecting shame, blame, and stigmatization identified as barriers in pre-COVID literature. Both primary prevention and survivor-centered support services, including those related to economic empowerment, should be integrated within COVID-recovery efforts, and extended into the post-pandemic period to fully meet women’s safety needs.

## Background

Gender-based violence (GBV) affects an estimated one in three women in her lifetime [1], with consequences including injury and death [2]. Over a third of homicides to women are committed by an intimate partner [3]. While intimate partner violence (IPV) is a leading form of GBV, other household members and individuals can also perpetrate emotional, physical, and sexual abuse with similarly negative impact on health and well-being [4, 5]. IPV can co-occur with household abuse to amplify risk and impacts [6–8].

Crisis and its aftermath increase risk for GBV, while undermining women’s economic and social standing [9, 10]. The COVID-19 pandemic raised global concerns for GBV [10, 11]. Available evidence demonstrates increases in GBV since COVID-19 in many settings [12], likely reflecting economic disruption, limited mobility, social isolation, increased time with potential abusers, financial and social stress, and new challenges to help-seeking.

GBV-related indicators, i.e., those that monitor GBV trends, must include both prevalence, and implementation and uptake of evidence-based GBV prevention and response (e.g., access to and use of violence support services). Disclosing abuse and obtaining safety planning and support is beneficial for survivors [13–15], yet violence support services are limited in many settings, and women often hide abuse due to shame, self-blame, impunity, and lack of knowledge of services [5]. Pandemic-related government-imposed mobility restrictions and fears of disease transmission can pose additional barriers to violence-related support services [11], further limiting access to care.

In Kenya and Burkina Faso, as in many settings, GBV was prevalent prior to the COVID-19 pandemic, with past-year IPV reported by 33% of ever-married women in Kenya (2014) [16] and 13% of partnered women in Burkina Faso (2010) [17]. Among 100 studies published on violence against women related to the COVID-19 pandemic, several studies have been conducted in Kenya [2]; results are mixed and include increases in both household tension and conflict, and increases in violence outside the home [18]. No results are currently available from Burkina Faso. To our knowledge, ours is the first study to focus on experiences of both IPV and household violence in Kenya and Burkina Faso during COVID-19 with population-based sampling.

We characterize: 1) prevalence of past-year IPV and other household violence, respectively; 2) changes in abuse frequency relative to the COVID-19 pandemic; and 3) associations of individual, dyad, and COVID-related factors with COVID-related abuse frequency; in two socially and culturally diverse settings highly affected by GBV—Kenya and Burkina Faso. Results provide timely evidence to guide GBV supports during the remainder of the pandemic, recovery investments that respond to safety needs, and insight into violence-related patterns for future emergencies. GBV evidence and evidence-driven prevention remain longstanding global priorities, articulated in the groundbreaking 1995 Beijing Declaration and Platform for Action, and reinvigorated 25 years later through the global Generation Equality movement initiated in 2020 to catalyze new progress towards the Beijing Platform’s goals.

## Methods

### Settings

Kenya and Burkina Faso have similar gender equity profiles; in 2019, both countries ranked in the lower half on the United Nations Development Program (UNDP) Gender Inequality Index (Burkina Faso 0.594; rank 147; Kenya 0.518, rank 126). Both ratified the Convention on the Elimination of all Forms of Discrimination Against Women (CEDAW), and have legal frameworks that criminalize domestic violence, however, implementation of social protection and access to justice remain challenging and IPV remains highly stigmatized. Both countries share a commitment to evidence-based violence prevention and response; the GBV survey module described herein was included at the request of in-country stakeholders and policymakers, including the Ministry of Health.

The national response to COVID-19 in Burkina Faso began on March 9, 2020, managed by the Centre des Opérations de Réponse aux Urgences Sanitaires (Ouagadougou, Burkina Faso), and primarily focused on physical distancing measures. Health services including GBV supports remained open throughout the pandemic; however, fear of infection decreased demand for services and prompted government-initiated radio messages to alert the public of service availability. In Kenya, the first case of COVID-19 was identified on March 13, 2020, and business and school closures were swiftly implemented, along with local curfews. GBV supports remained open and remotely accessible; the Kenyan government began to investigate reports of rising GBV cases in 2020, following the reported increases in case calls to the national domestic violence hotline between February and June, 2020 [19].

### Sampling

Performance Monitoring for Action (PMA) conducts annual population-based cross-sectional and panel surveys at the household, female, and service delivery levels. A multi-stage cluster sampling approach with probability-proportional-to-size sampling of enumeration areas produces nationally or regionally representative estimates. Further details are available at pmadata.org.

The present study utilizes cross-sectional female data collected in Kenya (November–December 2020) and Burkina Faso (December 2020–March 2021). Eligible study participants include females aged 15–49 within selected households. For respondent safety, only one woman per household was eligible to complete the GBV module, selected randomly via Open Data Kit (ODK) software in cases of multiple eligible participants.

### Ethical protections

Procedures followed best practices for violence research [20], and were approved by ethical review committees at Johns Hopkins School of Public Health, Kenyatta National Hospital-University of Nairobi Ethics and Research Committee College of Health Sciences in Kenya and Comite D’Ethique Pour La Recherche en Sante, Ministere de la Recherche Scientifique et de L’Innovation, Ministere de la Sante in Burkina Faso. Resident enumerators (REs) received GBV-specific training on confidentiality and privacy, non-judgmental questions, monitoring for emotional upset, and referral to support services. Privacy checks ensured that women completed sensitive questions in private. All female participants were given resource information, inclusive of GBV supports, reproductive health, and COVID-related resources.

### Analytic samples

**Fig. 1:**
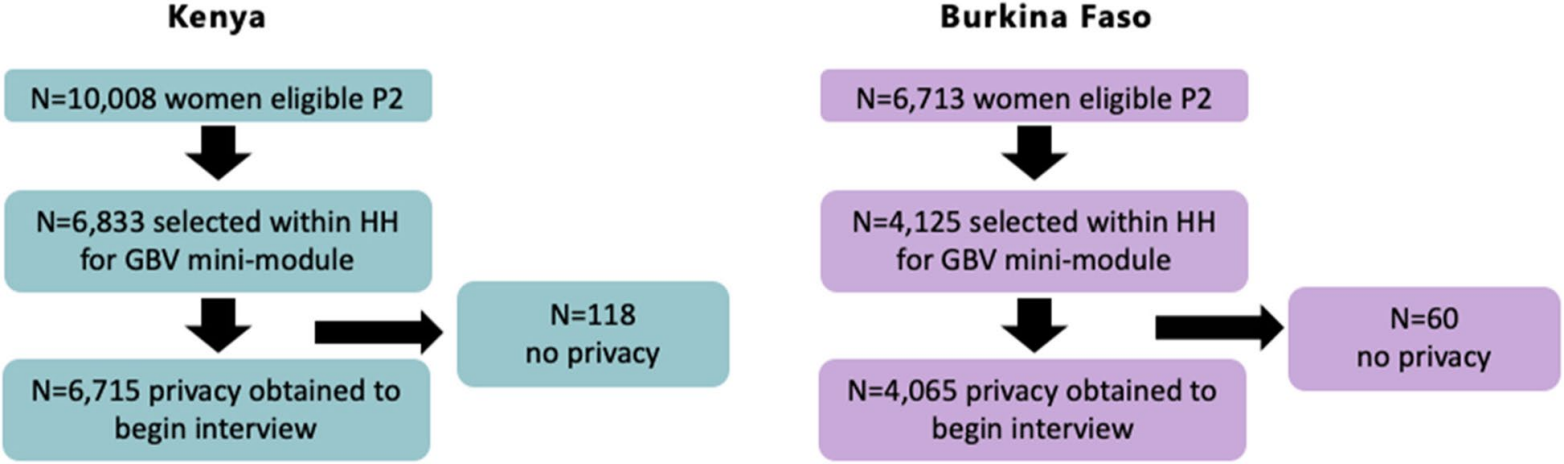
Flow Diagram of GBV Module Samples in Kenya and Burkina Faso

In Kenya, 10,008 women were eligible for the GBV module, and 6713 women in Burkina Faso. Random selection within households identified women to complete the GBV module (*n* = 6833 in Kenya; *n* = 4125 in Burkina Faso). Several did not complete the module due to privacy issues (Kenya *n* = 118; Burkina Faso *n* = 60), for a final sample of 6715 women in Kenya and 4065 women in Burkina Faso (Fig. 1).

All women selected for the GBV module received household violence questions; only women who were married/living with a partner completed the IPV portion (*n* = 4355 Kenya; *n* = 3048 Burkina Faso). The analytic samples used for multivariable models float to accommodate small amounts of missing covariate data (< 1%).

### Measures

Past-year IPV was measured via standard items rooted in the Revised Conflict Tactics Scale [21], indicated by an affirmative response to any of the following behaviors by a husband/partner: 1) Insulted you, yelled at you, screamed or made humiliating remarks, 2) Slapped, hit, or physically hurt you, 3) Threatened with a weapon or attempted to strangle or kill you, 4) Pressured or insisted on having sex when you did not want to (without physical force), 5) Physically forced you to have sex when you did not want to. IPV behaviors were examined individually and combined into sub-forms: item 1 (emotional violence), items 2–3 (physical violence), items 4–5 (sexual violence).

For indicated IPV behavior(s), single item(s) assessed frequency (Response categories: one time, 1–2 times, 3 to 10 times, more than 10 times, every day or almost), and changes in frequency relative to COVID-19 restrictions (more frequent, less frequent, or about the same).

Identical procedures assessed past-year household violence sub-forms, frequency, and COVID-related frequency, specifying that the behavior was by a “member of your household that is not your spouse or partner.”

Among those experiencing any IPV or household violence in the past 12 months, help-seeking was assessed via a single item: “Thinking about the experiences of relationship conflict we have just discussed, have you tried to seek help in the last 12 months?”; those indicating help sought were additionally asked, “From whom have you sought help?”

Additional domains included sociodemographic factors (age, marital status, education, residence, household wealth tertile, parity, number of household members, and residence with or without extended family). Economic factors include has savings, has mobile money account(s), level of financial knowledge (response on 4-point Likert scale and categorized as 0 = not knowledgeable at all, 1 = not very knowledgeable, 2 = somewhat or very knowledgeable), knows where to go for financial advice, and is working towards financial goals. COVID factors comprised concern with getting infected with COVID (dichotomized as not concerned/a little concerned vs. concerned/very concerned) and income loss in the past 12 months (none, partial, complete).

### Statistical analysis

Sample characteristics were described for women who participated in the GBV module and among partnered women, per setting. All violence outcomes were characterized by perpetrator (IPV, household), by setting (Kenya, Burkina Faso). The prevalence of each violence outcome was calculated overall, by item, and by violence sub-form (emotional, physical, sexual). Among women reporting violence, mean intensity of each item, change in frequency of each item during the COVID-19 restrictions, and help-seeking (overall and by violence type) were calculated.

Among partnered women, Venn diagrams were constructed to visualize overlap of IPV and household violence. Separate multivariable logistic regression models were used to examine correlates of past-year IPV or household violence experience, per setting; covariates significantly related at *p* < 0.1 from the bivariate models were included within the multivariable models (specified per model in table footnotes).

COVID-related frequency (i.e., overall changes to violence frequency in relation to COVID-19 restrictions—decreased, sustained, or increased) was characterized based on the following sequential decision-making rules: 1) if two forms of violence were indicated at the same frequency, COVID-related frequency took that frequency; 2) if any form of violence increased, COVID-related frequency is indicated as increased; 3) if one form of violence sustained and the other decreased, COVID-related frequency is indicated as sustained. Post-hoc analyses explored the potential for escalating forms of violence; by violence type, matrices were generated to explore escalation, i.e., substitution of one form for a more severe form.

Multinomial logistic regression models were then used to examine correlates of COVID-related frequency among those reporting violence (referent = decrease), per violence outcome and setting; measures with *p* < 0.1 from setting and outcome specific bivariate models were included within the final model, with only significant correlates reported in final tables. All analyses were conducted in STATA version 16 (College Station, TX), and weighted to account for the complex survey design.

## Results

Demographic characteristics were similar between contexts, withstanding education, where 59.2% of Burkinabe women never attended school, compared to over half (51.2%) of Kenyan women with at least secondary education (Table 1). Similarly, women’s reported economic standing was higher in Kenya than Burkina Faso, as evidenced by higher proportions of work outside the household (49.0% vs. 32.5%), savings (41.8% vs. 14.7%) and mobile money (69.6% vs. 25.5%) accounts, and levels of financial knowledge (75.1% vs. 7.2% very/somewhat knowledgeable). Over one in four women (26.0%) in Kenya reported complete income loss in the last year, compared to 9.4% in Burkina Faso, however, larger proportions of Burkinabe women attributed their income loss to COVID-19 restrictions (15.4% Burkina Faso vs. 6.9% Kenya). In both settings, most women who experienced income loss had partially recovered (63.5–64.9%), however, nearly one in three had not recovered (30.0% Burkina Faso; 32.3% Kenya).

**Table 1:** Demographic characteristics of women participating in the GBV Module by country

	Burkina Faso		Kenya	
	All women (*n* = 4065)	Married women; IPV sample (*n* = 3048)	All women (*n* = 6715)	Married women); IPV sample (*n* = 4355)
	%^a^			
**Sociodemographic**				
Residence				
Rural	76.8	80.4	68.2	69.4
Urban	23.2	19.6	31.8	30.6
Household wealth				
Lowest	33.6	35.1	34.6	37.0
Middle	32.5	33.8	34.3	33.6
Highest	33.9	31.1	31.1	29.5
Number of HH members				
1–2	5.1	4.4	10.2	6.8
3–4	26.6	28.1	34.6	38.5
5–7	37.4	37.9	42.7	44.4
8+	30.9	29.6	12.5	10.3
Household composition: Respondent				
Lives alone	0.5	0.2	2.7	0.5
Lives just with partner	3.0	3.7	3.3	5.1
Lives with nuclear family	54.6	59.9	60.0	70.4
Lives with extended family	41.8	38.0	34.1	23.9
Marital Status				
Married	74.3	90.9	59.0	89.5
Living with partner	7.1	9.1	5.53	10.5
Divorced/ Separated	1.3	–	5.89	–
Widow/ Widower	2.2	–	2.59	–
Never married	15.1	–	27.0	–
Age				
15–19	17.4	7.5	16.9	2.1
20–29	38.0	42.0	35.5	38.7
30–39	29.2	34.0	30.3	39.0
40–49	15.5	16.4	17.3	20.3
Education				
None	59.2	66.9	3.5	4.8
Primary	18.5	18.5	45.3	51.4
Secondary or Higher	22.4	14.4	51.2	43.9
Parity				
0	17.7	4.7	23.0	3.7
1–2	29.2	33.2	33.5	37.9
3+	53.1	62.2	43.5	58.5
**Economic**				
Works outside the HH, last 7 days	32.5	33.4	49.0	52.4
Works outside the HH, last 12 months	54.6	55.9	60.5	64.6
Paid for work				
No	21.7	21.4	10.4	9.6
In cash	70.8	71.0	77.7	77.6
In cash and in kind	4.4	4.6	9.9	11.0
In kind only	2.8	2.8	2.0	1.9
Has savings	14.7	15.3	41.8	48.0
Has mpesa mobile	25.5	24.2	69.6	76.4
Level of financial knowledge				
Not knowledgeable at all	76.1	74.6	5.1	3.3
Not very knowledgeable	16.7	18.2	19.8	18.5
Somewhat knowledgeable	5.3	5.4	37.9	39.2
Very knowledgeable	1.9	1.7	37.2	39.0
Knows where to get financial advice	20.9	21.8	48.2	51.6
Working towards financial goals	71.5	74.0	76.2	80.8
Economic dependence on partner	–	51.8	–	61.8
**Relationship Dyad**				
Husband’s nights away from home in last 12 months	–		–	
0	–	50.2	–	56.6
Less than 30 nights away	–	23.1	–	24.8
30 or more nights away	–	26.7	–	18.7
Partner education	–		–	
None	–	62.5	–	3.9
Primary	–	21.2	–	44.7
Secondary or Higher	–	16.3	–	51.4
Age at marriage	–		–	
≦15	–	5.3	–	7.0
> 15 & < 18	–	47.0	–	22.6
≧18	–	47.8	–	70.4
Husband has other partners	–		–	
Does not know	–	0.2	–	0.3
Yes	–	30.5	–	12.1
No	–	69.4	–	87.6
Financial Decision-Making Index (Scale 0–5)	–		–	
Decision-making score as mean (SD)	–	2.60 (1.54)	–	2.36 (1.49)
**COVID impact**				
Concerned with getting infected with COVID				
Not concerned	3.7	3.1	2.8	2.7
A little concerned	8.2	7.2	3.6	3.6
Concerned	16.7	16.3	20.6	20.5
Very concerned	71.4	73.5	73.0	73.1
I was infected with COVID	–	–	–	0.1
Income loss in the last 12 months				
None	53.9	52.9	21.2	18.6
Partial	36.7	37.2	52.8	54.9
Complete	9.4	9.9	26.0	26.6
Income loss in the last 12 months was from COVID restrictions (*n* = 2143, those who reported partial or complete income loss)				
No	84.6	84.9	93.2	93.6
Yes	15.4	15.1	6.9	6.4
Income partially or fully recovered in the last 4 weeks (*n* = 2142 [1mis], those who reported partial or complete income loss)				
Not recovered	30.0	30.9	32.3	32.1
Yes, partially	63.5	62.9	64.9	65.0
Yes, fully	6.5	6.1	2.8	2.91

^a^weighted

*=*p*<0.05; **=*p*<0.01; ***=*p*<0.001

**Fig. 2:**
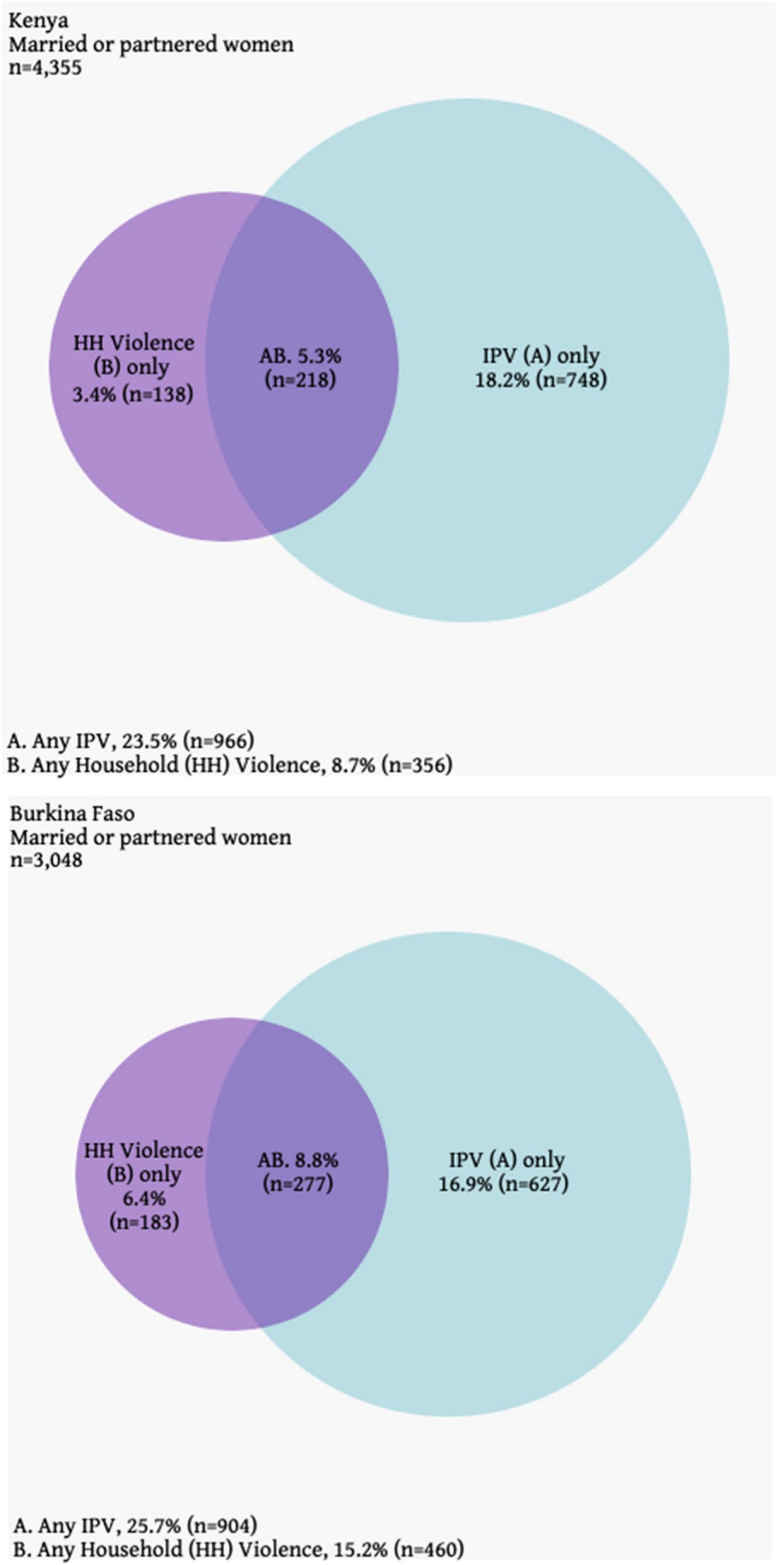
Venn Diagrams of past-year Household Violence and IPV Experience, Per Country, Among Currently-Partnered Women

Past-year IPV was experienced by approximately one in four women in both Burkina Faso (25.7%) and Kenya (23.5%; Table 2); past-year contact IPV (physical or sexual) was approximately one in ten (9.4% Burkina Faso; 13.4% Kenya). In both settings, past-year household violence prevalence was substantially lower, at 16.2% in Burkina Faso and 11.0% in Kenya, including for contact violence only (2.2% Burkina Faso; 4.7% Kenya). For both violence types and across settings, most women (51.8–89.0%) experienced a singular subset of violence; specifically, prevalence concentrated around emotional violence, with most women saying that this violence occurred between 1 and 10 times in the past year. Among partnered women, most experienced IPV only (18.2% Burkina Faso; 16.9% Kenya), relative to household violence only (3.4% Burkina Faso; 6.4% Kenya); approximately one in ten (8.8%) in Kenya and one in five (5.3%) in Burkina Faso experienced both IPV and household violence (Fig. 2).

**Table 2:** Past-year prevalence and intensity of IPV and non-partner household violence, and related help-seeking, per country

	Household Violence (Non-Partner)		IPV	
	Burkina Faso	Kenya	Burkina Faso	Kenya
**Past-year prevalence**	% weighted			
Any emotional	15.7	9.8	22.9	20.6
Any physical	1.7	3.7	4.5	8.6
Any sexual	0.6	1.7	6.4	8.0
Any violence (emotional, physical, sexual)	16.2	11.0	25.7	23.5
Any contact violence (physical, sexual)	2.2	4.7	9.4	13.4
**Types of violence experienced**				
Violence Score (# of specific types of violence experienced, includes emotional)^a^				
1 type of violence	89.0	66.2	71.8	51.8
2 types of violence	7.6	22.2	16.9	24.9
3 types of violence	3.3	6.6	7.7	14.1
4 types of violence	0.2	2.4	2.3	5.0
5 types of violence	–	2.6	1.3	4.2
**Violence intensity**^b^	% weighted, Mean (SD)			
Items: Prevalence & Frequency				
Insulted, yelled at, screamed at or made humiliating remarks	15.7, *2.56 (1.22)*	9.8, *2.09 (1.07)*	22.9, *2.66 (1.12)*	20.6, *2.39 (1.05)*
Slapped, hit, or physically hurt	1.3, *2.16 (1.17)*	2.7, *2.06 (1.03)*	4.2, *2.19 (1.13)*	7.4, *2.25 (1.02)*
Threatened with a weapon or attempted to strangle or kill	0.7, *2.50 (1.04)*	1.9, *2.15 (1.05)*	1.1, *2.28 (1.22)*	3.5, *2.35 (1.05)*
Pressured or insisted on having sex when did not want to (without physical force)	0.5, *2.26 (0.96)*	1.3, *2.12 (1.01)*	6.2, *2.48 (1.01)*	7.4, *2.37 (0.96)*
Physically forced to have sex when they did not want to	0.4, *2.17 (1.11)*	1.1, *2.16 (0.94)*	2.7, *2.14 (1.02)*	4.7, *2.38 (0.95)*
**Help-seeking, among those who indicated a violence experience**^c^	% weighted			
Any formal help	0.9	8.9	0.5	4.7
Any informal help	31.8	43.4	32.1	37.0
Did not seek help	67.9	51.6	67.8	60.6
**Sought help from:**				
Own family	41.5	57.8	42.7	58.9
Husband’s/partner’s family	41.2	26.8	45.9	41.2
Friend	26.4	24.4	2.0	2.9
Current/former husband/partner	5.5	5.2	0.2	0.0
Neighbor	4.7	18.5	26.3	21.3
Religious Leader	2.9	11.7	5.0	19.6
Police	2.0	14.9	5.4	16.3
Current/former boyfriend	0.7	2.3	1.4	2.2
Social service organization	0.7	3.3	1.2	7.7
Doctor/medical personnel	–	2.9	–	0.2
Lawyer	–	0.3	1.0	3.4
Violence support program or hotline	–	0.3	–	–

^a^Among those who reported any violence in the last 12 months

^b^Mean (SD) code: 1 = One time, 2 = 1 to 2 times, 3 = 3 to 10 times, 4 = 10 or more times, 5 = Every day or almost

^c^Formal and informal help categories are not mutually exclusive

*=*p*<0.05; **=*p*<0.01; ***=*p*<0.001

Across settings, over half of women did not seek help for the violence they experienced (Table 2). Any help-seeking was similar for household violence and IPV in Burkina Faso (32.1 and 32.2%, respectively), however, in Kenya, more women sought help for household violence than IPV (48.4% vs. 39.4%, respectively). Across violence types, help-seeking concentrated on informal help (31.8–43.4%), with the woman’s own family, the husband/partner’s family, or friends reported as most frequently sought sources.

Among women who experienced household violence, 14.3% of Burkinabe experienced increases in violence since COVID-19 restrictions, 26.9% reported unchanged levels, whereas 58.8% women reported decreases. In Kenya, 26.2% experienced increased violence since COVID-19, 29.3% unchanged, 44.6% experienced decreases. Relative frequency of IPV experiences since COVID-19 restrictions followed similar trends: in Burkina Faso, 64.8% decreased, 19.2% sustained, and 16.0% increased, and in Kenya, 36.4% decreased, 30.0% sustained, and 33.6% increased.

Post-hoc analyses tabulated changes in frequency by violence form to explore the potential for COVID-related escalation of violence type (e.g., substitution of emotional violence with physical violence); no evidence of substitution was detected.

### Associations with household abuse

In Burkina Faso, past-year household violence was associated with partial income loss in the past 12 months, compared to no income loss (aOR = 1.64; 95% CI = 1.16–2.30; Table 3). Additionally, all age groups less than 40 years old displayed increased odds of past-year household violence, compared to those 40–49 (aOR_15–19_ = 2.17, 95% CI = 1.27–3.70; aOR_20–29_ = 2.17, 95% CI = 1.33–3.54; aOR_30–39_ = 1.73, 95% CI = 1.01–2.95).

**Table 3:** Multivariable logistic and multinomial regression models for past-year household violence and changes in frequency relative to COVID-19 (referent = decreased household violence), per country

	Burkina Faso							Kenya						
	Multivariable Logistic		Multivariable Multinomial					Multivariable Logistic		Multivariable Multinomial				
	Any Household Violence (*n* = 710; 16.2%)		Decreased (ref) (*n* = 426; 58.8%)	Increased (*n* = 87; 14.3%)		Sustained (*n* = 173; 26.9%)		Any Household Violence (*n* = 701; 11.0%)		Decreased (ref) (*n* = 313; 44.6%)	Increased (*n* = 179; 26.2%)		Sustained (*n* = 207; 29.3%)	
	%*	OR (95% CI)	%*	%*	RRR (95% CI)	%*	RRR (95% CI)	%*	OR (95% CI)	%*	%*	RRR (95% CI)	%*	RRR (95% CI)
**Age and Household Structure**														
Age														
15–19	18.1	**2.17** (1.27, 3.70)^**^	59.3	14.6	1.48 (0.30, 7.38)	26.1	2.14 (0.55, 8.34)	17.0		40.9	28.0		31.1	
20–29	16.9	**2.17** (1.33, 3.54)^**^	53.9	17.0	2.22 (0.62, 7.99)	29.1	**3.45** (1.10, 10.76)^*^	10.1		47.4	22.8		29.8	
30–39	16.8	**1.73** (1.01, 2.95)^*^	56.8	13.1	1.77 (0.42, 7.41)	30.2	**3.35** (1.32, 8.45)^*^	8.9		40.9	29.8		29.3	
40–49	11.4	ref	81.0	8.0	ref	11.1	ref	10.9		49.9	24.7		25.4	
Marital Status														
Married	15.3		58.3	14.9	ref	26.8	ref	8.7	ref	49.1	25.3		25.6	
Living with partner	14.5		63.2	14.8	0.89 (0.30, 2.70)	22.0	1.02 (0.25, 4.18)	8.1	0.88 (0.54, 1.43)	56.5	25.0		18.5	
Divorced/separated or widower	18.7		79.3	3.3	0.23 (0.05, 1.12)	17.5	0.85 (0.21, 3.37)	16.5	**2.02** (1.47, 2.76)^***^	39.4	35.6		25.0	
Never married	21.0		54.7	14.6	0.84 (0.29, 2.45)	30.8	**2.63** (1.18, 5.86)^*^	15.1	**1.73** (1.21, 2.45)^**^	39.2	24.2		36.6	
Lives with extended family														
No	14.8		61.0	11.4		27.6		9.4	ref	46.6	24.3		29.0	
Yes	18.1		56.2	17.7		26.1		14.2	**1.34** (1.09, 1.67)^**^	41.9	28.5		29.6	
**Socioeconomic**														
Education														
None	15.4		60.5	10.2		29.4		13.2	1.18 (0.73, 1.92)	54.6	29.0		16.3	
Primary	18.4		56.7	19.6		23.8		12.9	ref	45.3	27.5		27.2	
Secondary or Higher	16.7		56.5	19.9		23.6		9.5	**0.73** (0.58, 0.92)^**^	42.4	24.6		33.0	
Household Wealth Tertile														
Lowest	14.8		63.2	5.1	ref	31.7	ref	14.2	ref	42.2	29.2		28.6	
Middle	17.1		56.7	17.2	**3.64** (1.17, 11.34)^*^	26.1	0.97 (0.48, 1.96)	11.3	0.85 (0.68, 1.07)	47.8	22.6		29.6	
Highest	16.7		56.8	19.8	3.57 (0.73, 17.42)	23.4	0.82 (0.35, 1.92)	7.2	**0.67** (0.49, 0.91)^*^	44.3	25.7		30.1	
Financial knowledge														
None/low	15.5		63.1	12.9		24.0		14.4		38.0	30.6	ref	31.4	ref
Medium	17.6		47.1	17.7		35.1		10.6		43.0	28.4	0.86 (0.40, 1.85)	28.6	0.95 (0.53, 1.70)
High	20.8		47.1	19.2		33.7		9.1		53.3	18.9	**0.47** (0.25, 0.87)^*^	27.8	0.75 (0.41, 1.37)
Income loss, past year														
None	13.5	ref	64.4	13.1	ref	22.4	ref	7.8	ref	52.1	23.2		24.7	
Partial	19.9	**1.64** (1.16, 2.30)^**^	51.9	16.2	1.23 (0.50, 3.00)	31.9	**1.80** (1.08, 2.98)^*^	10.5	**1.82** (1.30, 2.54)^***^	45.8	26.5		27.8	
Complete	16.8	1.37 (0.78, 2.42)	63.9	11.3	0.73 (0.19, 2.79)	24.8	1.17 (0.46, 3.00)	14.7	**2.58** (1.82, 3.67)^***^	39.6	27.0		33.4	

Multivariable logistic regression compares any household violence experience to no household violence experience (referent). Multivariable multinomial regression compares household violence trajectory since COVID-19 restrictions (increased, sustained) to decreased violence experience (referent). Model adjusted for all variables with *p* < 0.1 from bivariate models (not presented). Associations that remained significant (*p* < 0.05) after adjustment are presented. Burkina Faso logistic model adjusted for age, marital status, parity, household composition, and income loss in the last 12 months. Burkina Faso multinomial model adjusted for age, marital status, education, household wealth tertile, level of financial knowledge, and income loss in the last 12 months. Kenya logistic model adjusted for age, marital status, education, residence, parity, number of household members, household composition, household wealth tertile, savings, mobile money account, level of financial knowledge, knows where to go for financial advice, and income loss in the last 12 months. Kenya multinomial model adjusted for marital status, parity, level of financial knowledge, knows where to go for financial advice, and has financial goal

*=*p*<0.05; **=*p*<0.01; ***=*p*<0.001

In multinomial models, increases in household violence frequency since COVID-19 were seen for women within the middle wealth tertiles, compared to lowest tertile (aRRR = 3.64; 95% CI = 1.17–11.34). Sustained experiences of household violence since COVID-19  were associated with middle age groups (aRRR_20–29_ = 3.45; 95% CI = 1.10–10.76; aRRR_30–39_ = 3.35; 95% CI = 1.32–8.45), never being married (aRRR = 2.63; 95% CI = 1.18–5.86) or suffering partial income loss in the last 12 months (aRRR = 1.80; 95% CI = 1.08–2.98).

In Kenya, past-year experience of household violence was associated with past-year income loss (aOR_partial_ = 1.82, 95% CI = 1.30–2.54; aOR_complete_ = 2.58, 95% CI = 1.82–3.67), being divorced, separated, or widowed (aOR = 2.02; 95% CI = 1.47–2.76), never being married (aOR = 1.73; 95% CI = 1.21–2.45), and living with extended family (aOR = 1.34; 95% CI = 1.09–1.67; Table 3). Protective factors included highest wealth groups (aOR = 0.67; 95% CI = 0.49–0.91) and secondary or higher education (aOR = 0.73; 95% CI = 0.58–0.92). Within multivariable multinomial models, only high financial knowledge was protective against increased household violence since COVID-19 (aRRR = 0.47; 95% CI = 0.25–0.87).

### Associations with IPV

In Burkina Faso, past-year IPV was associated with husband spending less than 30 nights away from home in the past year (aOR = 1.47; 95% CI = 1.09–1.97); economic reliance on the husband/partner for basic needs was protective (aOR = 0.65; 95% CI = 0.49–0.87; Table 4).

**Table 4:** Multivariable logistic and multinomial regression models for past-year IPV and changes in frequency relative to COVID-19 (referent = decreased IPV), per country

	Burkina Faso							Kenya						
	Multivariable Logistic		Multivariable Multinomial					Multivariable Logistic		Multivariable Multinomial				
	Any IPV (*n* = 904; 25.7%)		Decreased (ref) (*n* = 542; 64.8%)	Increased (*n* = 165; 16.0%)		Sustained (*n* = 175; 19.2%)		Any IPV (*n* = 966; 23.5%)		Decreased (ref) (*n* = 367; 36.4%)	Increased (n = 313; 33.6%)		Sustained (*n* = 286; 30.0%)	
	%*	OR (95% CI)	%*	%*	RRR (95% CI)	%*	RRR (95% CI)	%*	OR (95% CI)	%*	%*	RRR (95% CI)	%*	RRR (95% CI)
Residence														
Urban	29.0		53.8	22.3	ref	24.0	ref	22.6		27.9	37.7	ref	34.5	ref
Rural	24.9		67.9	14.3	0.53 (0.27, 1.06)	17.8	**0.34** (0.13, 0.88)^*^	23.9		39.9	31.9	0.70 (0.41, 1.19)	28.2	**0.55** (0.30, 1.00)^*^
**Partner dyad**														
Husband’s nights away from home in the past 12 months														
0 night away	24.3	ref	62.0	18.2		19.8		20.7	ref	33.1	32.0		35.0	
Less than 30	31.6	**1.47** (1.09, 1.97)^*^	64.3	14.3		21.4		30.2	**1.65** (1.31, 2.09)^***^	40.1	34.6		25.4	
30 or more	23.6	0.96 (0.70, 1.33)	70.0	14.4		15.6		23.4	1.17 (0.90, 1.54)	38.8	36.4		24.8	
Partner education														
None	25.5		63.8	16.0	ref	20.2	ref	39.6		61.8	25.5		12.7	
Primary	27.2		61.6	17.6	0.85 (0.48, 1.53)	20.9	1.00 (0.62, 1.60)	27.2		35.6	33.6		30.8	
Secondary or Higher	24.6		73.7	14.1	**0.37** (0.19, 0.74)^**^	12.2	**0.47** (0.25, 0.90)^*^	19.1		33.4	34.9		31.7	
Husband has other wives														
No	25.6		64.0	18.5		20.2		22.1	ref	35.0	35.0		30.0	
Yes	25.8		66.8	16.4		16.8		33.8	**1.48** (1.13, 1.94)^**^	42.8	27.0		30.0	
Decision-making score														
≤ 3	23.4		66.3	17.1		16.6		27.3	ref	40.6	32.7		26.7	
> 3	31.6		61.9	14.0		24.2		21.9	**0.79** (0.64, 0.98)^*^	34.1	34.1		31.8	
Economically reliant on husband/partner for basic needs														
No	30.2	ref	64.4	15.9		19.7		25.1		35.2	30.6		34.3	
Yes	21.5	**0.65** (0.49, 0.87)^**^	65.4	16.1		18.5		22.6		37.2	35.7		27.1	
**Socioeconomic**														
Education														
None	24.1		66.5	14.1	ref	19.4	ref	31.8		59.7	26.8	0.81 (0.30, 2.15)	13.6	0.58 (0.22, 1.56)
Primary	28.1		54.6	18.8	1.39 (0.73, 2.63)	26.7	1.62 (0.94, 2.79)	27.1		33.1	34.3	ref	32.6	ref
Secondary or Higher	30.0		70.7	19.8	1.25 (0.51, 3.11)	9.5	**0.44** (0.22, 0.89)^*^	19.0		37.6	34.0	**0.67** (0.46, 0.97)^*^	28.4	0.67 (0.42, 1.06)
Household Wealth														
Lowest	22.0		67.4	6.6	ref	26.1	ref	29.0		38.2	31.5		30.3	
Middle	27.3		66.4	20.0	**3.36** (1.66, 3.78)^**^	13.6	**0.46** (0.23, 0.92)^*^	22.3		38.5	30.5		31.0	
Highest	28.1		60.9	20.4	**3.33** (1.26, 8.82)^*^	18.7	0.56 (0.20, 1.55)	18.1		29.8	42.2		28.1	
Income loss, past year														
None	22.5		66.5	13.3		20.3		17.4	ref	43.6	20.9	ref	35.5	ref
Partial	28.1		63.3	18.6		18.1		22.3	**1.68** (1.19, 2.38)^**^	36.4	32.7	1.55 (0.88, 2.72)	30.9	0.84 (0.42, 1.67)
Complete	33.4		63.7	17.5		18.8		30.4	**2.38** (1.66, 3.42)^***^	33.4	40.1	**2.03** (1.05, 3.92)^*^	26.5	0.70 (0.35, 1.42)

Multivariable logistic regression compares any IPV experience to no IPV experience (referent). Multivariable multinomial regression compares IPV experience trajectory since COVID-19 restrictions (increased, sustained) to decreased IPV experience (referent). Model adjusted for all variables with *p* < 0.1 from bivariate models (not presented). Associations that remained significant (*p* < 0.05) after adjustment are presented. Burkina Faso logistic model adjusted for age, education, parity, household wealth tertile, worked in the last year, decision spending score, and income loss in the last 12 months. Burkina Faso multinomial model adjusted for age, education, residence, parity, household wealth tertile, level of financial knowledge, knows where to go for financial advice, and partner education. Kenya logistic model adjusted for education, household wealth tertile, savings, mobile money account, knows where to go for financial advice, financial goal, husband's nights away from home, partner education, husband has other wives, decision spending score, and income loss in last 12 months. Kenya multinomial model adjusted for education, residence, household wealth tertile, work last year, mobile money account, financial goal, husband's nights away from home, partner education, husband has other wives, decision spending score, and income loss in last 12 months. ^* ^*p*<0.05^*^

In multinomial models, increased frequency since COVID-19 was observed for women in the middle (aRRR = 3.36; 95% CI = 1.66–3.78) and highest (aRRR = 3.33; 95% CI = 1.26–8.82) household wealth tertiles, whereas partner having attained secondary or higher education was protective (aRRR = 0.37; 95% CI=0.19-0.74 ). Protective factors for sustained COVID-related frequency include secondary or higher education (aRRR = 0.44; 95% CI = 0.22–0.89), rural residence (aRRR = 0.34; 95% CI = 0.13–0.88), middle wealth tertile (aRRR = 0.46; 95% CI = 0.23–0.92), partner attending secondary or higher education (aRRR = 0.47; 95% CI = 0.25–0.90).

In Kenya, past-year IPV was associated with having husband spent less than 30 nights away from home in the past year (aOR = 1.65; 95% CI = 1.31–2.09), polygyny (aOR = 1.48; 95% CI = 1.13–1.94), and partial or complete income loss during COVID-19 (aOR_partial_ = 1.68, 95% CI = 1.19–2.38; aOR_complete_ = 2.38, 95% CI=1.66–3.42). Conversely, higher decision-making autonomy was protective (aOR = 0.79; 95% CI = 0.64–0.98).

Increased IPV frequency since COVID-19 was associated with complete loss of income in the past year (aRRR = 2.03; 95% CI = 1.05–3.92); secondary or higher education was protective (aRRR = 0.67; 95% CI = 0.46–0.97).

## Discussion

Past-year IPV was prevalent for women in Kenya and Burkina Faso; estimates exceeded those for household violence. During the COVID-19 pandemic, substantial proportions of affected women experienced sustained or increased frequency of abuse, though decreases in frequency were also noted. This comprehensive study includes two leading forms of violence (household and IPV) across two distinct sites, enabling understanding of factors that may be context specific (i.e., financial autonomy indicators) vs. more universal (i.e., COVID-related income loss) in their impact. Psychological abuse was prominent, and even at lower levels of intensity, is linked with health consequences [22]; accordingly, monitoring efforts to understand COVID-impact must extend beyond physical and/or sexual violence. The low levels of help-seeking, particularly for formal supports, are concerning yet consistent with pre-pandemic global trends. Improving access to and use of GBV-related support services, including for emotional abuse, is highly actionable through public health messaging that educates and normalizes support service use. In a global dialogue focused on increased GBV during COVID-19, results add important nuance to changes in violence dynamics prior to and through early stages of the pandemic, and affirm the need for sustainable prevention and response following pandemic recovery. Results provide important new learning in two priority settings. Specifically, in Burkina Faso, results fill a dearth of evidence on violence against women during COVID-19. In Kenya, results advance a growing evidence base by providing necessary clarification on the nature of abuse (household vs. IPV) and timing relative to the pandemic. Results affirm risk of violence to women from both partners and other household members during public health emergencies.

Economic factors were linked with experiences of IPV and household abuse across settings, though with contextual variation. Specifically, recent income loss increased risk for both forms of abuse. Past-year income loss increased risk for household violence (partial income loss only for Burkina Faso; partial/complete income loss in Kenya). In Burkina Faso, this income loss was also linked with sustained levels of violence since the onset of the pandemic; income loss similarly increased IPV risk in Kenya. By contrast, household wealth tertiles diverged across sites in their associations with violence. In Kenya, higher household wealth *protected* against household violence, while in Burkina Faso, household wealth *elevated* risk for household violence and IPV since COVID-19. Prior population-based research has similarly found some forms of violence linked with greater wealth in Burkina Faso [23]. Notably, these indicators examine wealth at the household level via inventory of household assets and are not specific to women’s own wealth. While economic empowerment programs have evidenced both women’s and families’ benefit from accumulation of wealth at the household level, women’s own role in wealth generation and access to household assets likely vary and may account for discrepancy of findings.

Household abuse was shaped by power dynamics specific to age, marital status, and household structure. In Burkina Faso, past-year prevalence was highest for the youngest women and decreased with age. Marital status conferred some protection against household violence—never-married women in Burkina Faso had increased risk for sustained violence through COVID-19, and in Kenya, divorced/separated/widowed and never married women had increased risk for past-year household violence. Increased risk to women who lack the relative social protection of marriage is consistent with evidence from other settings [24]. The increased risk for household violence for women living in extended family households potentially reflects in-law abuse as has been found in other settings [4, 6–8]. Young women’s relatively greater burden may result from more limited leverage and relative power.

IPV patterns and risk sources diverged somewhat across sites, reflecting contextual differences in the influence of gendered systems that structure norms and autonomy. In Kenya, dyad-level risk factors for IPV included presence of other wives; by contrast in Burkina Faso, where polygyny is more normative, no such elevated risk was identified. In Kenya, higher financial decision-making scores were *protective* against past-year IPV; comparatively, in Burkina Faso, economic *reliance* on partners was protective. It is striking that indicators of financial independence and autonomy are protective against IPV in Kenya, where women’s financial autonomy is more normative, as evidenced by high levels of savings and financial knowledge. By contrast, in Burkina Faso, where norms are more aligned with traditional gender hierarchies, the economic reliance on partners confers protection against IPV. Other research has similarly found that the relationship of financial indicators to IPV is highly contextual [25].

Notably, violence-related help-seeking was low in both Burkina Faso and Kenya. Moreover, women heavily relied on informal supports, primarily family, despite the expansion of support services, judicial trainings, and awareness-raising activities in recent years. The reluctance to seek formal services is consistent with global evidence [5, 16], and may reflect social norms and gendered social systems that tolerate or minimize abuse and stigmatize those who share their experiences beyond family [5]. Because tolerance and stigma challenges women’s ability to seek help or identify their experiences as abuse [26, 27], services must communicate accessibility and confidentiality to overcome barriers to care-seeking.

Several limitations should be noted. Social desirability biases and privacy concerns could contribute to underreporting of abuse, particularly for more sensitive forms like sexual violence, despite extensive training and privacy protocols aligned with best practices. Recall bias and errors are possible, particularly regarding timing of experiences relative to the pandemic. To limit survey length, abbreviated measures were used. Household violence and IPV measures were designed for comparability; the household measure does not specify the perpetrator, which limits specificity for resulting programmatic recommendations. National level analysis may mask important within-country heterogeneity.

While alarming, the high prevalence of violence against women is highly actionable. World Health Organization guidelines for clinic-based violence assessment and response [28] can be embedded in COVID-related and post-pandemic response. Technology-based solutions for IPV safety assessment and planning have been effective in Kenya [29] and can be scaled; these offer accessibility advantages during mobility restrictions such as pandemics and future health emergencies. Economic empowerment programs can reduce risk [30]; these programs are particularly important given the pandemic's detrimental impact to women’s social and economic opportunity, though must be implemented with care to ensure success.

## Conclusions

The COVID-19 pandemic creates a window of opportunity for GBV policy and programming. Evidence that violence at the hands of partners and other household members in Kenya and Burkina Faso was prevalent both prior to and during the pandemic illustrates that the needs are not pandemic-specific; rather violence prevention and response must sustain into post-pandemic rebuilding. Governments must take swift action to prioritize gender equity, destigmatize violence, scale evidence-based prevention approaches, and normalize access to meaningful support for survivors. Essential steps include replacing violence-related silence and stigma with a culture of survivor-centered support. Doing so will advance the Sustainable Development Goal of elimination of violence against women, and generate cascade positive impact on women’s health and well-being.

## Data Availability

The datasets generated and analyzed during the current study are available from www.pmadata.org on reasonable request.

## Abbreviations

CEDAW: Convention the Elimination of all forms of Discrimination Against Women
GBV: Gender-based violence
IPV: Intimate partner violence
ODK: Open Data Kit
PMA: Performance Monitoring for Action
RE: Resident enumerator
UNDP: United Nations Development Program
